# Evaluation of the physicochemical, proximate, and sensory properties of moinmoin from blends of cowpea and water yam flour

**DOI:** 10.1002/fsn3.592

**Published:** 2018-04-10

**Authors:** Gloria Aderonke Otunola, Anthony Jide Afolayan

**Affiliations:** ^1^ Medicinal Plants and Economic Development (MPED) Research Center Department of Botany University of Fort Hare Alice South Africa

**Keywords:** cowpea, food security, *moinmoin*, protein–energy malnutrition, water yam

## Abstract

Moinmoin is a steamed cowpea seed‐based pudding native to Nigeria. This study evaluated the physicochemical, proximate, and sensory properties of moinmoin from a blend of cowpea and water yam flours. The pudding was prepared by varying the proportion of cowpea to water yam flour (CWYP). The blends were in the ratio 75:25% (CWYP1), 50:50% (CWYP2), and 100:0% cowpea flour (CPP) which served as the control. Physicochemical evaluation indicated that the products will have good keeping quality and reconstitute easily. Protein content was 26.90%, 20.10%, and 17.60% for CPP, CWYP1 and CWYP2, respectively, and the presence of water yam significantly (*p* < .05) increased the crude fiber, ash, and carbohydrate contents of the cowpea/water yam products relative to the control. Pasting properties revealed that regardless of the proportion of water yam in the mixture, the moinmoin samples cooked at approximately the same time; and sensory evaluation showed that CWYP1 was best preferred in terms of taste, flavor, color, and overall acceptability. These findings suggest that the cowpea/water yam pudding can meet the nutritional demands of a meal and that the 75%–25% cowpea/water yam mixture was the most acceptable. The study contributes to the knowledge nutritious products with desirable organoleptic qualities from a blend of cowpea and water yam flour, allowing for up to 50% water yam substitution. It also provides an alternative way of utilizing water yam thus preventing wastage during peak production and ensuring food and nutritional security because of the balanced products obtained.

## 1 INTRODUCTION

Food security exists when all people, at all times, have physical, social, and economic access to sufficient, safe, and nutritious food which meets their dietary needs and food preferences for an active and healthy life (FAO, IFAD, & WFP, 2014). Unfortunately however, in most developing countries of the world, hunger and malnutrition are on the rise due to increase in population, shortage of farmlands, and high food prices (FAO, 1990; Von Braun et al., 2008). Protein malnutrition is one of the major public health challenges throughout the developing world especially in many parts of Africa where the diets are predominantly starchy (Akusu & Kiin‐Kabari, 2012; Müller & Krawinkel, 2005; Olatidoye & Sobowale, 2011). This is because; the major food crops in these areas are cereals, roots, and tubers (Nnabuk, Ita, Dodo, & Paul, 2012; Olatidoye & Sobowale, 2011). Legumes are an excellent source of plant protein; contributing up to 18% of the world's protein requirement, dietary fiber, and mineral nutrients such as iron, potassium, folic acid, and zinc (Duranti, 2006; Olatidoye & Sobowale, 2011).

Leguminous species range from the well‐known crops such as cowpea (*Vignia unguiculata*) and soybean (*Glycine max*) to the lesser known and underutilized wing beans (*Psophocarpus tetragonlibas*). Their seeds are highly nutritious with high‐protein content, as such; they are used as food for both humans and animals. In addition, cowpea in particular is an important component of cropping systems in the tropics usually used as cover crops, green manure, and as natural fertilizers (Nwokolo, 1996). It is consumed in many forms such as the young leaves, green pods/seeds as vegetables, and dry seeds in various food preparations (Singh, Ehlers, Sharma, & Freire‐Filho, 2002). In West Africa, cowpea seeds are boiled with condiments and eaten alone with stew or in combinations with cereals. In Nigeria, they are also used in preparing other food products such as fried cowpea cakes, bean soup, and a steamed popular delicacy called *moinmoin*. Nutritionally, cowpea seeds contain 61%–66% carbohydrates, 24%–25% proteins, and 1.0%–2.0% lipids (Bressoni, 1985; Madode, Linnemann, Nout, Vosman, & Hounhouigan, 2012).

*Dioscorea* is a genus with over 600 species of flowering plants in the family Dioscoreaceae. The vast majority of the species are tropical with only a few extending into temperate climates. Several species, known as yams, are important agricultural crops in tropical regions, grown for their large tubers and are particularly important in parts of Africa, Asia, and Oceania. One of the edible species is *Dioscorea alata,* a tuberous root vegetable, usually bright white in color, also known as water yam. With its origins in the Asian tropics, *D. alata* has been known to humans since ancient times. The species is characterized by a high‐water content and not as sweet as the other edible species.

Nutritionally, water yam has 70%–73% moisture content, 19%–22% carbohydrate, 0.58%–1.0% fat, 6%–8% protein, 4%–5% ash, and a high content of the B vitamins and essential minerals (Wireko‐Manu, Ellis, Oduro, Asiedu, & Maziya‐Dixon, 2011). It has high yield, high multiplication ratio and better tuber storability than several other edible species. Unfortunately, *D. alata* has low economic value as it is less used for major yam products such as pounded yam, fufu, boiled or fried yam. This is because its flesh is watery and usually not as firm as those of other edible yams. However, *D. alata* has an advantage over others. It has higher sustainable cultivation ecology, especially nowadays when the production of other yams seems to be on the decline (Wireko‐Manu et al., 2011). Considering the poverty level and inadequate food supply as a result of increase in world population, the ecological adaption for easy and sustainable production of *D. alata* should be exploited for food security.

According to Singh et al. (2002), the addition of even a small amount of cowpea to cereals, roots, and tubers could enhance the nutritional balance of the diet and protein quality. This is achieved by the synergistic effect of high protein and lysine from cowpea and high methionine and energy from cereals.

*Moinmoin* is a traditional Nigerian bean dish obtained by steaming homogeneous slurries containing cowpea paste, small amounts of vegetable oil, pepper, and other ingredients. On steaming the slurry in pouches made from leaves, aluminum foil or polyethylene bags, it solidifies into an irreversible gel‐pudding (Akusu & Kiin‐Kabari, 2012; Okechukwu, Ngoddy, Nnanye‐lugo, & Nnanye‐lugo, 1992).

Galati, Oguntoyinbo, Moschetti, Crescimanno, and Settani (2014) reported that most people from Africa and other developing countries live on diets based mainly on cereals, roots, and tubers. These types of diet provide more than 60% of the total energy supply, with minimal or no protein of animal origin. Although these staples contribute significant amounts of fiber, minerals, and vitamins, they are limiting in some of the essential amino acids especially lysine; thus diets based on cereals, roots, and tubers alone are not sufficient in ensuring balanced diet. Also, because of the low‐protein content of cereals, roots, and tubers, protein–energy malnutrition is prevalent in populations where they are used as staples, especially among women and children.

Water yam (*D*. *alata*) is rich in cysteine, isoleucine, and lysine (Awoyale, Maziya‐Dixon, Sanni, & Shittu, 2016); while cowpeas are good sources of proteins and rich in lysine, though deficient in sulfur‐containing amino acids, such as methionine and cysteine. Blending or combining cereals, roots, and tubers with a protein‐rich source will contribute to the improvement of the nutritional quality of such foods (Liao et al., 2004). It follows then that by appropriately combining cowpea with water yam, a well‐balanced dish should result.

According to Chandra, Singh, and Kumari (2015), functional properties are the fundamental physicochemical properties that reflect the complex interaction between the composition, structure, molecular conformation, and physicochemical properties of food components together with the nature of environment in which these are associated and measured. Functional characteristics are important in determining product performance and have influence on sensory characteristics of food products. There has been little or no study on the functional and sensory properties of moinmoin made from blends of cowpea and water yam. Therefore, this study was conducted to assess the physicochemical, nutritional, and sensory characteristics of moinmoin products made from cowpea/water yam composite flours toward improving the nutritional quality and diversifying the food uses of water yam to prevent its wastage during peak productivity.

## 2 MATERIALS AND METHODS

### 2.1 Preparation of flour blends

Water yam tubers, cowpea seeds, and other ingredients were purchased from a local market in Ilorin, Nigeria. All reagents used in this study were of analytical grade.

Water yam flour was produced using the method described by Udensi, Oselebe, and Iweala (2008) with slight modifications. The tubers were washed, peeled, sliced, and blanched at 80°C for 4 min. They were dried, milled, and sieved through 100‐μm mesh.

Cowpea seeds were cleaned, sorted, and steeped in water for 2 hr, dehulled manually by rubbing between the palms and dried at 60°C for 3 hr in a hot air oven. This was then milled and sieved through 100‐μm mesh.

Flour blends of cowpea and water yam were prepared in ratios of 100:0, 75:25, and 50:50; and coded as CPP, CWYP_1_, and CWYP_2_), respectively. The flours were separately and thoroughly mixed at these ratios using a Kenwood food mixer to obtain homogeneous blends.

### 2.2 Formulation and preparation of moinmoin

Three blends of moinmoin from cowpea and water yam were prepared using the formulations shown in Table 1. Each blend was separately mixed with a wooden spoon until all the ingredients were well blended. The resultant blend was dispensed into small aluminum bowls used for pudding preparation and steamed for 45 min.

**Table 1:** Blends of cowpea/water yam flours and ingredients for *moinmoin* production

Samples	Water yam flour (g)	Cowpea flour (g)	Total (%)	Chili pepper (g)	Vegetable oil (g)	Salt (g)	Onion (g)	Stock cubes (g)
CPP	0.00	100.00	100.00	30.00	100.00	3.00	20.00	8.00
CWYP_1_	25.00	75.00	100.00	30.00	100.00	3.00	20.00	8.00
CWYP_2_	50.00	50.00	100.00	30.00	100.00	3.00	20.00	8.00

CPP, cowpea flour; CWYP, cowpea to water yam flour.

### 2.3 Physicochemical properties of flour blends

The pH of the samples was determined using a pH meter (Model 049016, Crison Instruments, S.A., Alella‐Barcelona, EU). The titratable acidity (TTA) was determined as previously described by Adeyemi and Oluwamukomi (2002), and the result expressed as percentage lactic acid. Water absorption capacity was measured as described by Chandra et al. (2015), while the reconstitution index was determined as previously described (Oluwatooyin, Osundahunsi, & Aworh, 2002; Onwuka & Ihuma, 2007). All determinations were in triplicates, and the mean value was recorded in each case.

### 2.4 Pasting properties of flour blends

Pasting viscosity of the flour blends was determined as described by AACC (2001) using a Rapid Viscosity Analyzer (RVA–1998, Model RVA‐SUPER3; Newport Scientific, Australia). Pasting temperature, peak viscosity temperature, peak viscosity during heating and viscosity on cooling to 50°C were recorded.

Briefly, 3 g of flour samples was weighed into a dry empty canister; 30 ml of water was added and thoroughly mixed before fitting into the visco analyzer. Each suspension was kept at 50°C, then heated up to 95°C at 12.2°C per min and held for 2.5 min at this temperature. The suspension was then cooled to 50°C at 12.0°C and held for 2 min at 50°C. All determinations were in triplicates, and the mean value was recorded in each case.

### 2.5 Proximate analysis

The moisture, crude protein, ash, crude fat, and crude fiber contents of the blends were determined using the AOAC (2002) methods. Carbohydrate content was determined by subtracting the values of the other components from 100. Each determination was in triplicate, and the mean value was recorded. Energy value was calculated using the Atwater conversion factors.

### 2.6 Sensory evaluation

Sensory properties of the moinmoin were determined by panels of 20 untrained people familiar with the product. The coded samples were presented in white plastic plates, and panelists were instructed to rinse their mouth with water between samples. The assessors were asked to appreciate how much they liked the taste, appearance, flavor, texture, and the overall acceptability of the moinmoin on a hedonic scale varying from 1 (dislike extremely) to 7 (like extremely).

### 2.7 Statistical analysis

Results were analyzed using Minitab^®^, version 12, software. Analysis of variance was used for data relating to sensory evaluation, and Duncan's Multiple Range Test was used to separate and compare the group means. Significance was accepted at *p* < .05.

## 3 RESULTS

### 3.1 Physicochemical properties of flour blends

Results for TTA, pH, water absorption capacity, and reconstitution index are presented in Table 2. The pH values of all the samples were in the acidic range but decreased slightly from control (CPP) to CWYP_2,_ while titratable acidity increased from 0.28 into 0.45. There were also significant (*p* < .05) differences in water absorption capacity and reconstitution index of CPP relative to CWYP_1_ and CWYP_2_ with the lowest reconstitution index value.

**Table 2:** Physicochemical properties of cowpea water yam flour blends

Physicochemical properties	CPP	CWYP_1_	CWYP_2_
pH	6.35^a^ ± 0.00	6.20^a^ ± 0.00	6.15^a^ ± 0.00
Titratable acidity (% LAE)	0.28^a^ ± 0.01	0.39^b^ ± 0.01	0.45^c^ ± 0.03
Water absorption capacity (%)	16.10^a^ ± 0.02	18.60^b^ ± 0.20	20.67^c^ ± 0.04
Reconstitution index (%)	27.90^a^ ± 0.02	25.60^b^ ± 0.00	22.40^c^ ± 0.02

CPP, cowpea flour; CWYP, cowpea to water yam flour; LAE, lactic acid equivalent.

Values are means of three determinations ± SD.

Values along the same row with different superscripts are significantly different.

### 3.2 Pasting properties of flour blends

**Figure 1:**
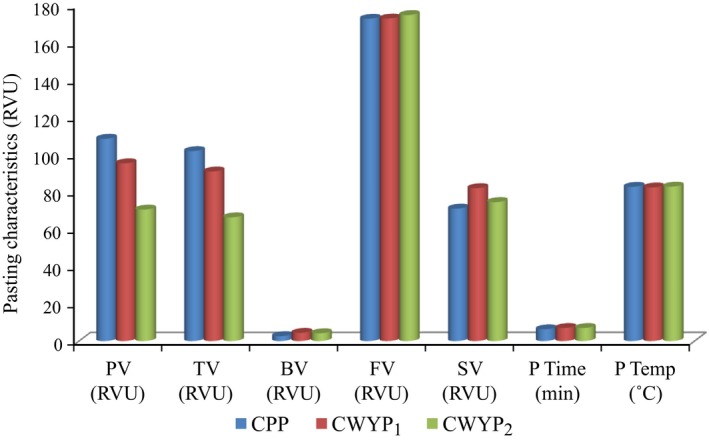
Pasting characteristics of cowpea/water yam flour blends. PV, pasting viscosity; TV, trough viscosity; BV, breakdown viscosity; FV, final viscosity; SV, setback value; PT, peak time; P temp, pasting temperature; RVU, rapid visco analyzer unit

The pasting properties of the flour blends are presented in Figure 1. There was a significant (*p* < .05) difference in the peak viscosity (PV) of the control when compared to the cowpea: water yam blends. The control (CPP) had the highest viscosity, followed by CWYP_1_ and CWYP_2_, respectively. CWYP_2_ exhibited the lowest trough viscosity, but increased final viscosity and peak temperature; while break down viscosity ranged from 2.63 in CPP to 4.38 in CWYP_1_. No significant (*p* > .05) difference in the final viscosity, set back value, peak time, and pasting temperature was observed in all the flour blends.

### 3.3 Proximate and Energy content of moinmoin from flour blends

The data obtained for the proximate analysis of the samples are presented in Table 3. There was a significant (*p* < .05) difference in the moisture contents of the pudding samples. CPP had the highest protein and lipid contents compared to the cowpea/water yam products. Sample CWYP_1_ had the highest moisture content, while sample CWYP_2_ had the highest carbohydrate and crude fiber contents. The estimated energy content of the puddings was 344.80, 331.10, and 325.40 kcal/100 g for CPP, CWYP_1_, and CWYP_2_, respectively.

**Table 3:** Nutrient content and estimated energy value of *moinmoin*

Nutrient (%)	CPP	CWYP_1_	CWYP_2_
Moisture	13.50^a^ ± 0.02	15.00^b^ ± 0.02	12.50^c^ ± 0.02
Protein	26.90^a^ ± 0.01	20.10^b^ ± 0.02	17.60^c^ ± 0.01
Lipid	10.00^a^ ± 0.01	9.50^b^ ± 0.01	9.00^b^ ± 0.01
Ash	6.50^a^ ± 0.02	7.30^b^ ± 0.01	9.00^c^ ± 0.01
Crude Fiber	6.30^a^ ± 0.01	6.80^b^ ± 0.01	8.40^b^ ± 0.01
Carbohydrate	36.80^a^ ± 0.02	41.30^b^ ± 0.02	43.50^c^ ± 0.01
Energy value (kcal/100 g)	344.80^a^ ± 1.93	331.1^b^ ± 1.07	325.4^c^ ± 0.92

CPP, cowpea flour; CWYP, cowpea to water yam flour.

Values are means of three determinations ± SD.

Means along the same row with different superscripts are significantly different.

### 3.4 Sensory evaluation of moinmoin prepared from cowpea/water yam blends

**Figure 2:**
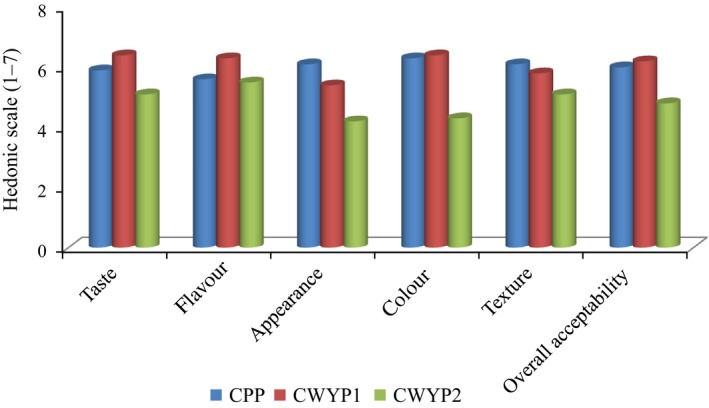
Sensory properties of *moinmoin* prepared from cowpea/water yam blends

Results of the organoleptic tests of the pudding using the flour blends are as presented in Figure 2. CWYP_1_ was rated highest in terms of taste, flavor, color, and overall acceptability. It was closely followed by CPP, while CWYP_2_ was the least accepted.

## 4 DISCUSSION

The acidity of flour is an important indication of its freshness. TTA and pH of the cowpea/water yam pudding flour samples revealed that addition of water yam did not lead to any significant increase in acidity. Low TTA ensures the proper absorption of mineral elements and is also an indication that the product will have better keeping quality because the acidity could prevent or delay the growth of spoilage microbes (Abioye, Ade‐Omowaye, Babarinde, & Adesigbin, 2011; Kuyunga, Mbugua, Kangethe, & Imungi, 2011). Water absorption capacity is an important functional property in food formulations required for bulking, consistency and in baking applications which affects the final product attributes such as flavor retention, mouth feel, and shelf life of products (Ikegwu, Okechukwu, Ngoddy, Nnanye‐lugo, & Ekumankana, 2010). The addition of water yam to cowpea led to an increase in the capacity of the blends to absorb water as shown by the high water absorption capacity. This could be attributed to the difference in protein concentrations and the degree of interaction of such proteins with water (Ikegwu et al., 2010). Similar increase in the water absorption capacities of cassava–sorghum flour meals, cocoyam–soybean–crayfish flour blends and composite flour from cassava, rice, potato, soybean, and xanthan gum has been reported (Oti & Akobundu, 2007; Perez & Perez, 2000; Tharise, Julianti, & Nurminah, 2014). Although all the samples easily reconstituted, a decrease in the reconstitution index with increasing proportion of water yam in the blend was observed. This is an indication that increasing the content of water yam in the blend increases the water absorption capacity which in turns improves the reconstitution ability of the flour blends. Similar observations have been reported (Adebowale, Sanni, & Onitilo, 2008; Adegunwa, Ganiyu, Bakare, & Adebowale, 2014).

Pasting properties influence quality and esthetic considerations in the food industry because they affect texture and digestibility as well as the end use of starch‐based foods (Adebowale, Sanni, & Awonorin, 2005; Ajanaku, Ajanaku, Edobor‐Osoh, & Nwinyi, 2012; Osungbaro, Jimoh, & Osundeyi, 2010). Pasting characteristics measure the viscosity of flours over a range of temperature and pressure and are used for predicting the ability of flour to form a paste when subjected to heat applications.

The peak viscosity is the highest viscosity reached during gelatinization; it reflects the ability of starch granules to swell freely before their physical breakdown during heating and is related to the water‐binding capacity of starch. The high peak viscosity displayed by the flour blends indicate that they are suitable for products requiring high gel strength and elasticity such as *moinmoin*. Trough viscosity, the point at which viscosity reaches the minimum during heating or cooling processes measures the ability of the gel to withstand breakdown during cooling. The significantly high trough viscosity observed in the 100% cowpea flour (CPP) and the 25:75% cowpea water yam blend indicates the tendency of the flours to breakdown during cooling and is similar to the values reported earlier (Danbaba et al., 2012). However, the final viscosity (FV) and the viscosity on cooling (PT) to 50°C showed a reverse trend, increasing with increase in the proportion of water yam flour. These observations suggest that the water absorption capacity of the water yam flour is far greater than that of the CPP as confirmed earlier in Table 3. In all cases, the final viscosities of the flours were greater than the peak viscosities, implying that even on cooling, the starches in the mixtures continued to absorb water and become more swollen, which might result in stiffer moinmoin from the blends.

Setback viscosity is the tendency of starch granules to retrograde on cooling. According to Iwe, Onyeukwu, and Agiriga (2016) the higher the setback viscosity, the lower the retrogradation of the flour paste during cooling and the lower the staling rate of the products made from the flour. In this study, the CWYP_1_ blend had the highest setback viscosity which implies that its products are likely to have a very good texture and not stale quickly. Both the pasting temperatures and peak times, which are indicators of the temperature at which gelatinization begins and the time to reach peak viscosity, did not present much variation, though the highest value was recorded for CWYP_2._ The implication of this is that regardless of the proportion of water yam, the mixture would cook at approximately the same time and at the same temperature as the control.

The moisture content of any food is an indicator of its water content, stability, and susceptibility to microbial contamination (Uyoh, Ita, & Nwofia, 2013). The highest moisture recorded for CWYP_1_ was still relatively low when compared to other studies (Davis et al., 1991; Ibeanu, Onyechi, Ani, & Ohia, 2016). The low moisture content of the moinmoin is an indication of storage stability and longer shelf life. The high‐protein content observed in the CPP moinmoin could be attributed to the significant quantity of protein (about 24%) in cowpea seeds (Jimoh & Olatidoye, 2009). The pudding with 25:75% cowpea water yam blend exhibited a higher protein and lipid content compared with the pudding with 50:50% cowpea water yam substitutions. The lower fat content of the moinmoin from cowpea/water yam blends will reduce the rate at which rancidity sets in subsequently increasing the shelf life. This also makes the products suitable for weight management. The presence of 75% and 50% contents of cowpea significantly increased the protein content of the water yam‐containing products (CWYP_1_ and CWYP_2_) considering that water yam has only 6%–8% protein content (Jimoh & Olatidoye, 2009). This expected increase was the basis for formulating the blends in order that the final products will have higher protein content and can thus be used to prevent malnutrition. Similar increase in protein content has been reported when cassava flour was supplemented with soy flour (Olatidoye & Sobowale, 2011) and when plantain flour was fortified with soy flour (Abioye et al., 2011). The two composite pudding products CWYP_1_ and CWYP_2_ also exhibited higher ash, crude fiber, and carbohydrate contents when compared with the CPP products as a result of the presence of water yam in the composite. The ash, crude fiber, and carbohydrate content increased with increasing level of water yam substitution. This high nutrient content of the blends will be of nutritional importance in most developing countries where many people can hardly afford high‐proteinous foods because of the high costs. The low‐energy level observed in the pudding products can be attributed to their low crude fat and high‐protein contents. This is an indication that the puddings are low‐calorie foods which may be very helpful in weight management.

Overall, proximate values for all the samples showed that the three products irrespective of the water yam content have high nutritional quality and low‐energy values.

Sensory evaluation showed that both CPP and CWYP_1_ had similar overall acceptability. This is an indication that the addition of 75% cowpea to 25% water yam gave a product that is acceptable both organoleptically and nutritionally as the 100% cowpea pudding. Sample CWYP_2_ was least acceptable probably because of the high proportion of the water yam in it which darkened the color, making it unattractive and rendering its taste much less acceptable. The result of the sensory evaluation revealed that pudding from cowpea paste alone and that produced from the addition of 75:25 cowpea water yam was rated alike in almost all the quality attributes evaluated indicating the acceptability of moinmoin from a blend of cowpea and water yam. These blends are suitable for combating protein–energy malnutrition in a predominantly starch‐ and cereal‐based diet; and their consumption can suppress nutrient deficiencies as a viable long‐term, food‐based, strategy to control nutrient deficiencies.

## 5 CONCLUSION

This study has revealed that addition of cowpea to water yam flour in the proportion of 75% or 50% gives a product that is nutritionally acceptable and organoleptically better (25% substitution only) than the well‐known moimoin from cowpea alone. The 100% moinmoin (control) and that of water yam: cowpea 25:75% (CWYP1) was rated alike in almost all the quality attributes.

While water yam is abundant, it is less utilized for major food products as a result of traditional bias which fails to recognize its unique quality characteristics. It can be inferred therefore that water yam/cowpea pudding could be an acceptable addition to the menu especially in regions where PEM is prevalent and both crops are produced in abundance. It also provides an alternative way of utilizing water yam thus preventing wastage during peak production and ensuring food security. This is especially important because yam production seems to be on the decline as a result of high cost of production, low yields, and postharvest losses.

## CONFLICT OF INTEREST

The authors declare no conflict of interest.
